# Supplementary material for: New Chalcone Derivatives Containing 2,4-Dichlorobenzenesulfonamide Moiety with Anticancer and Antioxidant Properties
Source: Int J Mol Sci. 2023 Dec 24;25(1):274. doi: 10.3390/ijms25010274 (PMC10778824; doi:10.3390/ijms25010274)
Supplement: Supplementary file 1 [file ijms-25-00274-s001.zip › ijms-2763236-supplementary.pdf]

# Supplementary material

# New Chalcone Derivatives Containing 2,4-dichlorobenzenesulfonamide Moiety with Anticancer and Antioxidant Properties

**Anita Bułakowska[1,*], Jarosław Sławiński[1], Anna Hering[2], Magdalena Gucwa[2], J. Renata Ochocka[2], Rafał Hałasa[3], Justyna Stefanowicz-Hajduk[2,*]**

[1] Department of Organic Chemistry, Medical University of Gdańsk, Al. Gen. J. Hallera 107, 80-416 Gdańsk, Poland, jaroslaw@gumed.edu.pl (J.S.), bulanit@gumed.edu.pl (A.B.)

[2] Department of Biology and Pharmaceutical Botany, Medical University of Gdańsk, Al. Gen. J. Hallera 107, 80-416 Gdańsk, Poland, jadwiga.ochocka@gumed.edu.pl (J.R.O), justyna.stefanowicz-hajduk@gumed.edu.pl (J.S.-H.), anna.hering@gumed.edu.pl (A.H.)

[3] Department of Pharmaceutical Microbiology, Medical University of Gdańsk, Al. Gen. J. Hallera 107, 80-416, Gdańsk, Poland, rafal.halasa@gumed.edu.pl (H.R.)

* Correspondence: bulanit@gumed.edu.pl, justyna.stefanowicz-hajduk@gumed.edu.pl

**Tabele of contents**

**Appendix A:**

**Spectrum 1.** $^1$H NMR of compd **3** (500 MHz, DMSO-$d_6$).

**Spectrum 2.** $^1$H NMR of compd **4** (500 MHz, DMSO-$d_6$).

**Spectrum 3.** $^{13}$C NMR of compd **4** (125 MHz, DMSO-$d_6$)

**Spectrum 4.** $^1$H NMR of compd **5** (500 MHz, DMSO-$d_6$).

**Spectrum 5.** $^{13}$C NMR of compd **5** (125 MHz, DMSO-$d_6$).

**Spectrum 6.** $^1$H NMR of compd **6** (500 MHz, DMSO-$d_6$).

**Spectrum 7.** $^{13}$C NMR of compd **6** (125 MHz, DMSO-$d_6$).

**Spectrum 8.** $^1$H NMR of compd **7** (500 MHz, DMSO-$d_6$).

**Spectrum 9.** $^{13}$C NMR of compd **7** (125 MHz, DMSO-$d_6$).

**Spectrum 10.** $^1$H NMR of compd **8** (500 MHz, DMSO-$d_6$).

**Spectrum 11.** $^{13}$C NMR of compd **8** (125 MHz, DMSO-$d_6$).

**Spectrum 12.** MS spectrum of compound **4**, MS (ESI) $m/z$ = 444.

**Spectrum 13.** MS spectrum of compound **5**, MS (ESI) $m/z$ = 522.

**Spectrum 14.** MS spectrum of compound **6**, MS (ESI) $m/z$ = 462.

**Spectrum 15.** MS spectrum of compound **7**, MS (ESI) $m/z$ = 478.

**Spectrum 16.** MS spectrum of compound **8**, MS (ESI) $m/z$ = 458.

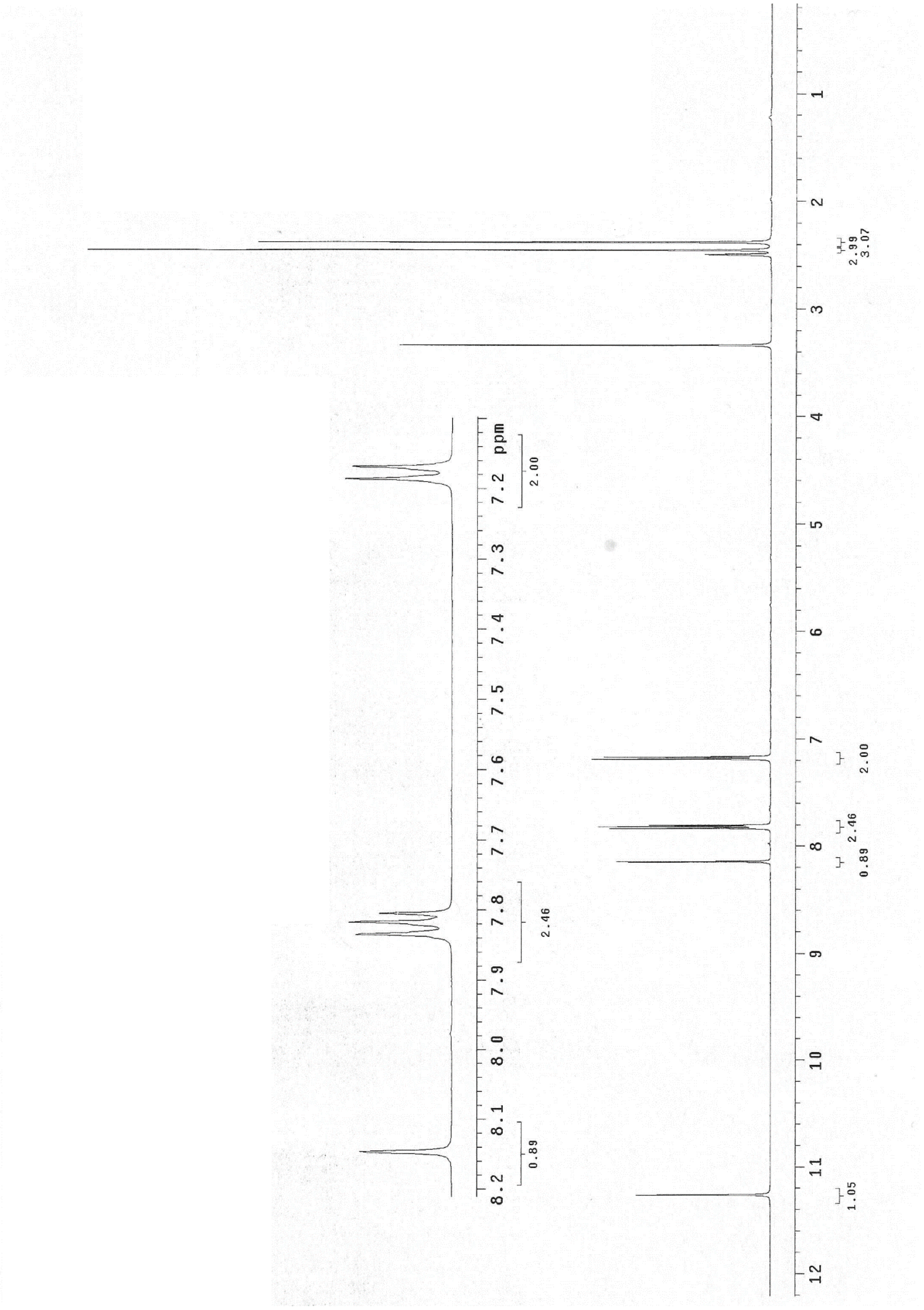


**Spectrum 1**. $^1$H NMR of compd **3** (500 MHz, DMSO-$d_6$).

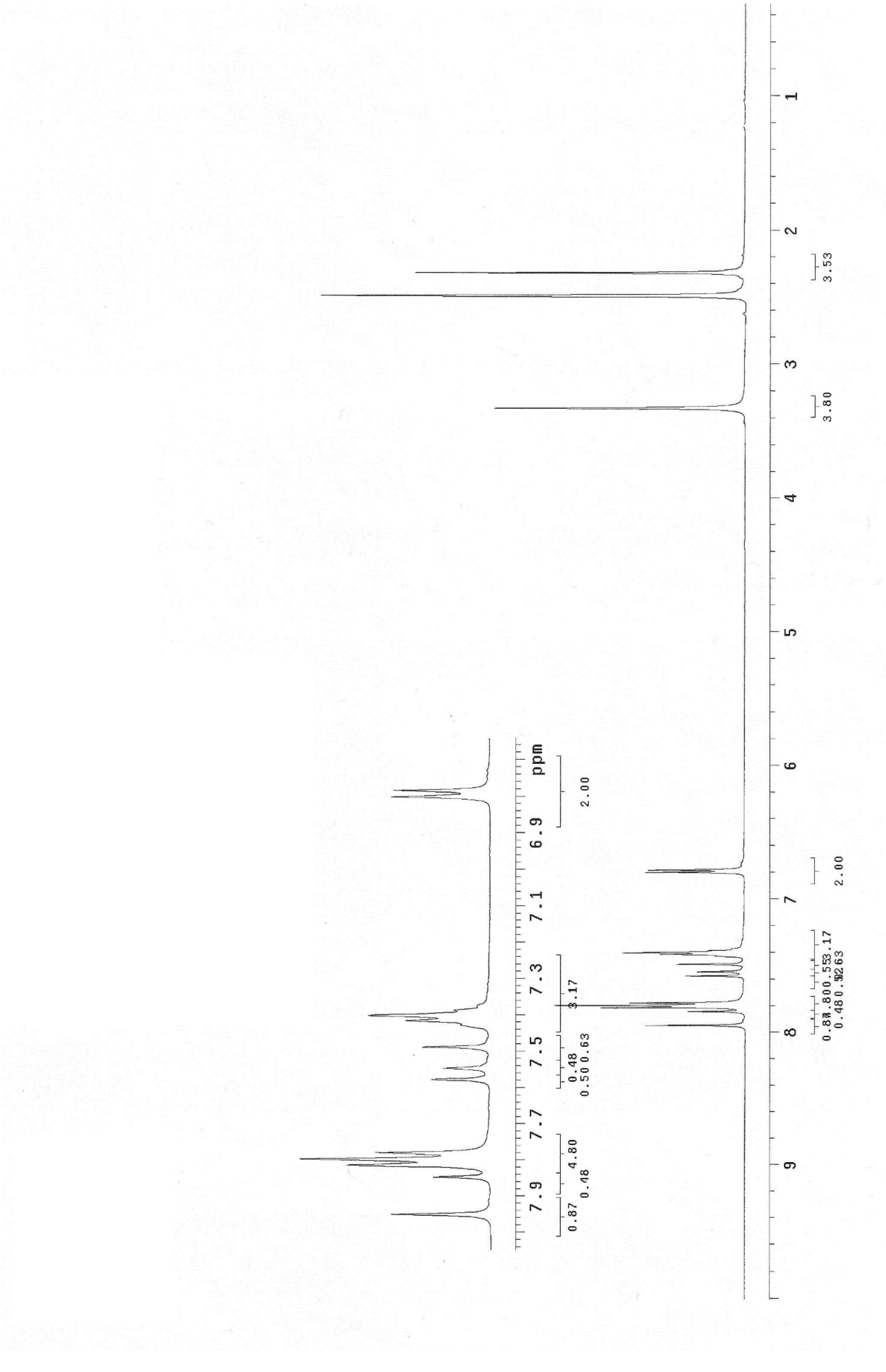


**Spectrum 2**. $^{1}$H NMR of compd **4** (500 MHz, DMSO-$d_6$).

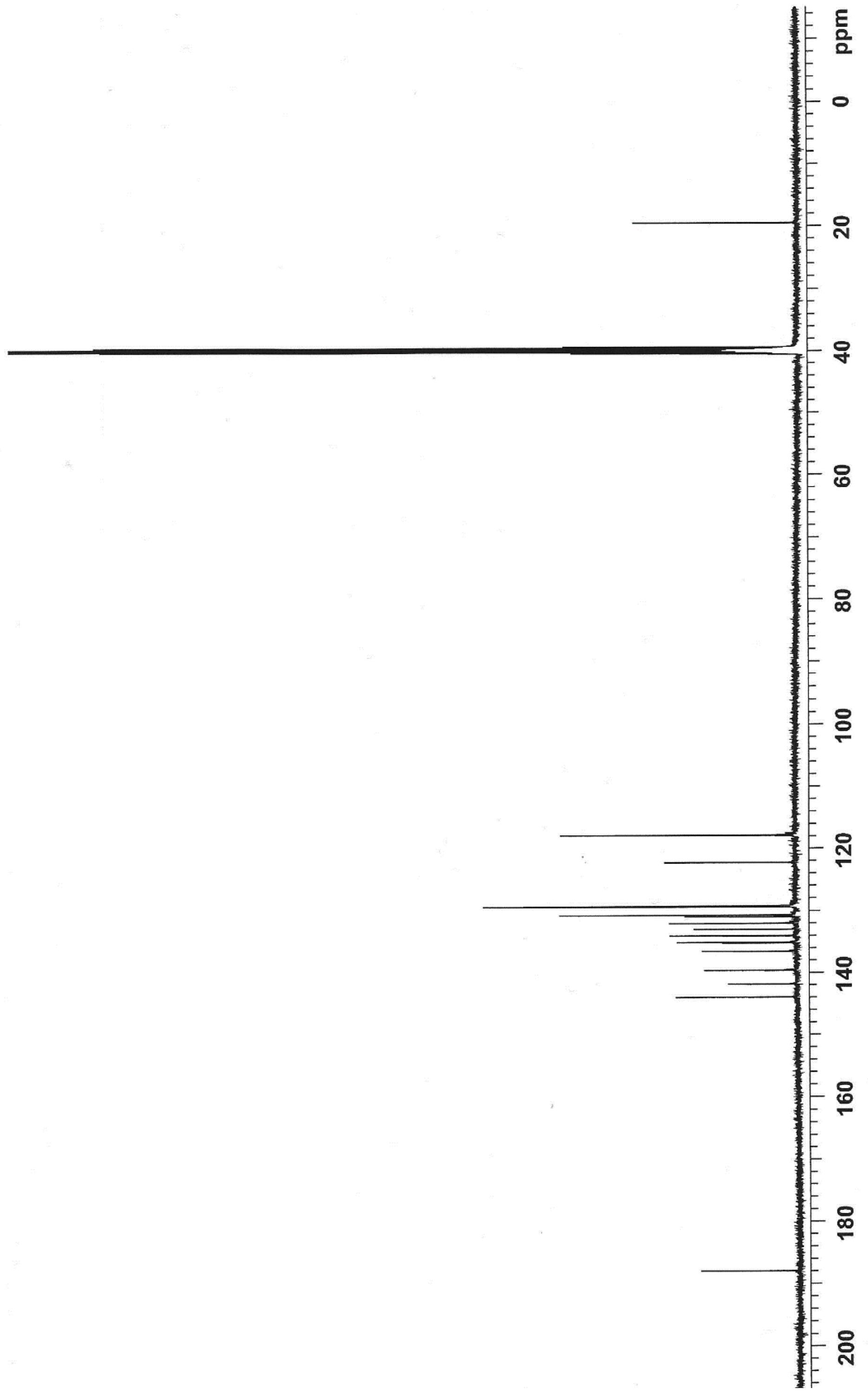


**Spectrum 3**. $^{13}C$ NMR of compd **4** (125 MHz, DMSO-$d_6$).

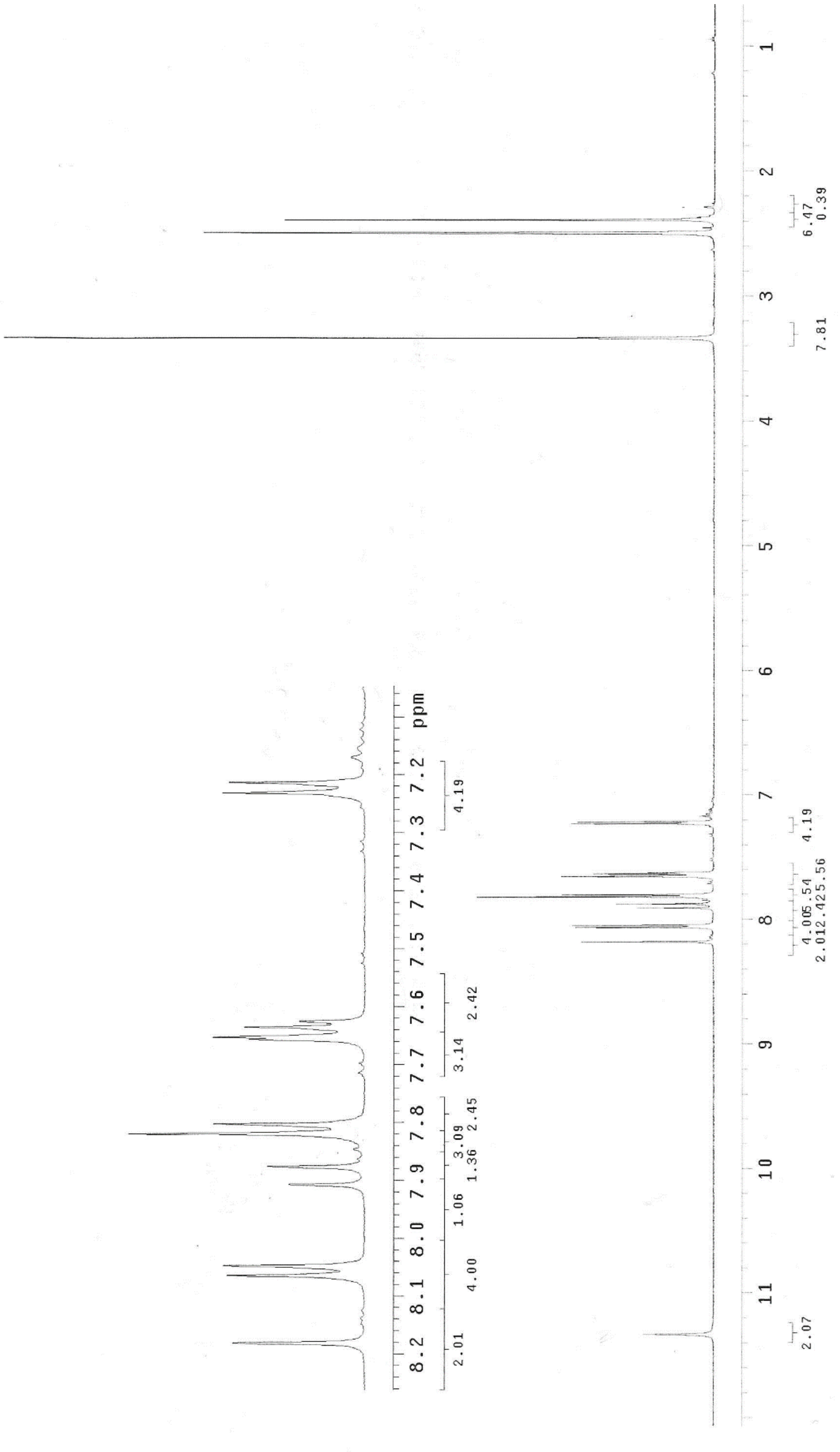

**Spectrum 4**. $^{1}$H NMR of compd **5** (500 MHz, DMSO-$d_6$).

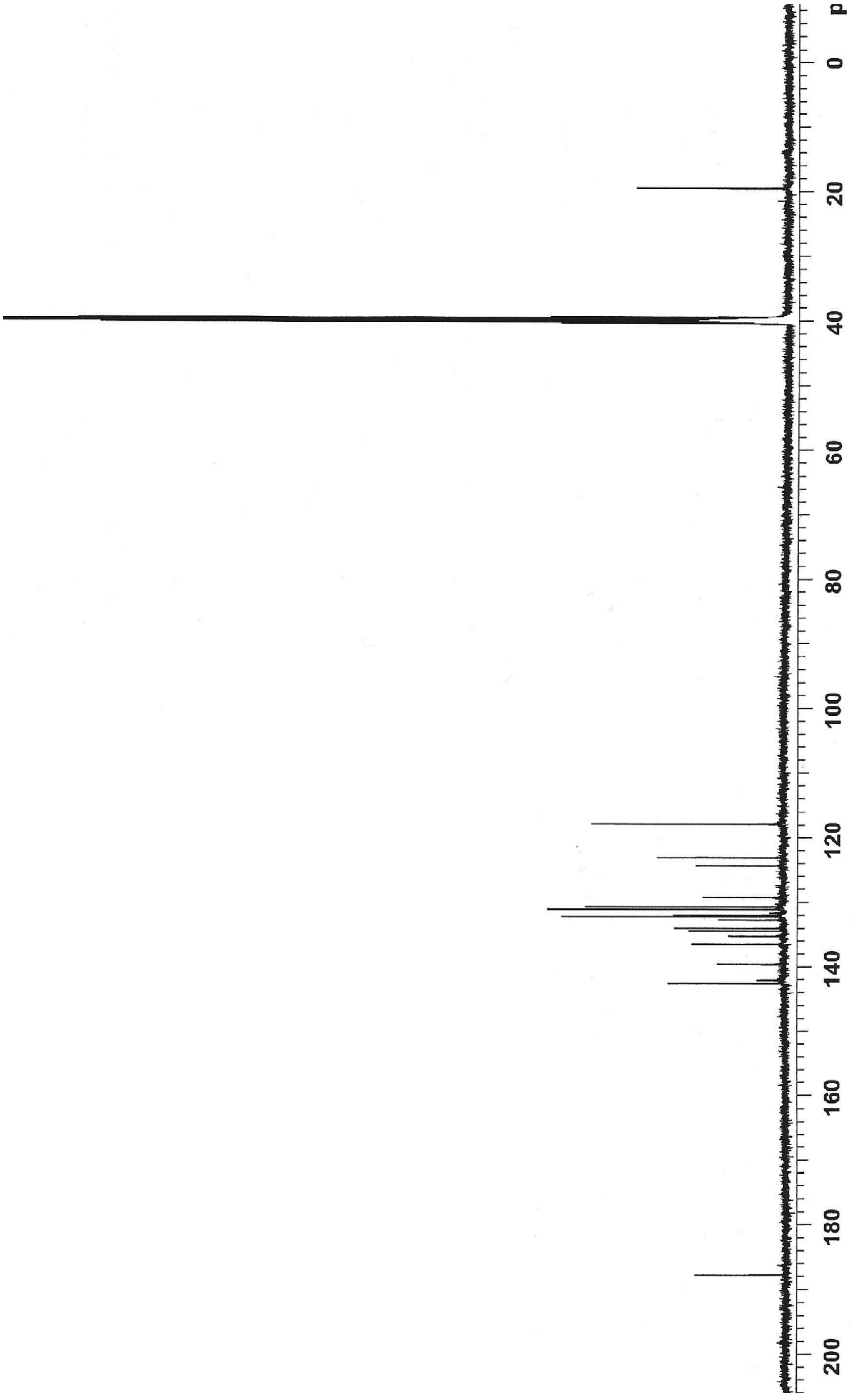


**Spectrum 5**. $^{13}C$ NMR of compd **5** (125 MHz, DMSO-$d_6$).

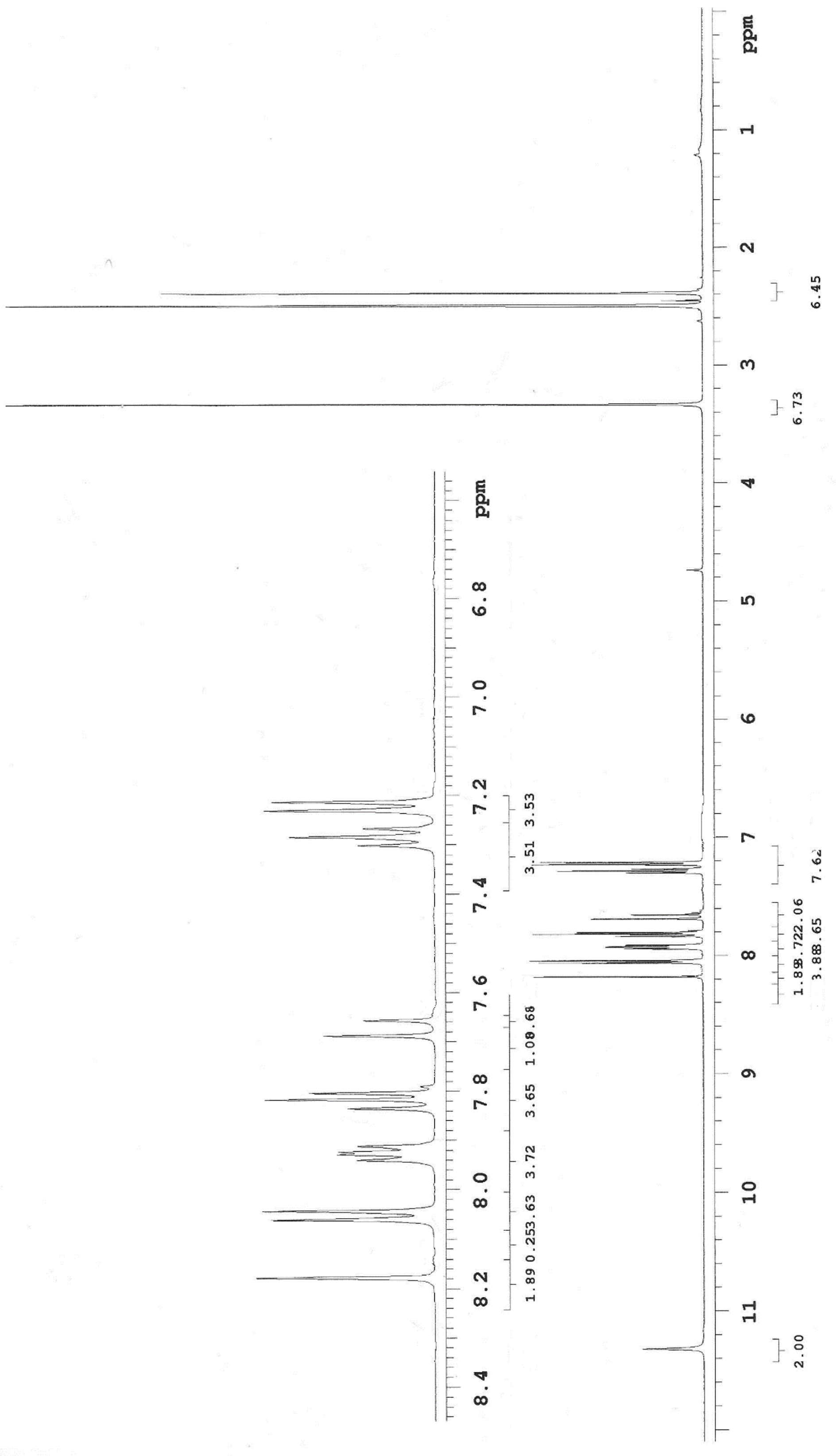


**Spectrum 6**. $^{1}$H NMR of compd **6** (500 MHz, DMSO-$d_6$).

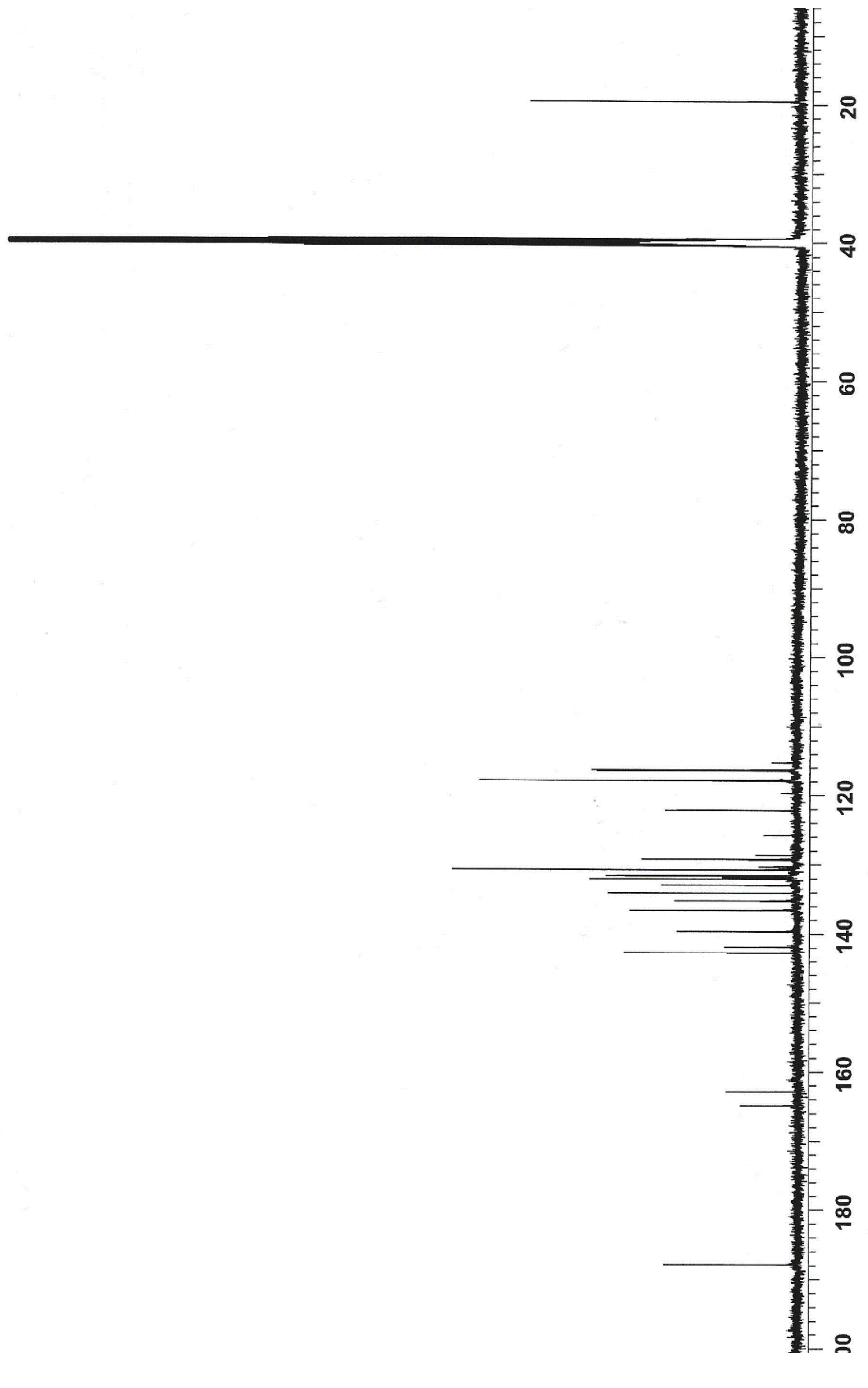


**Spectrum 7**. $^{13}C$ NMR of compd **6** (125 MHz, DMSO-$d_6$).

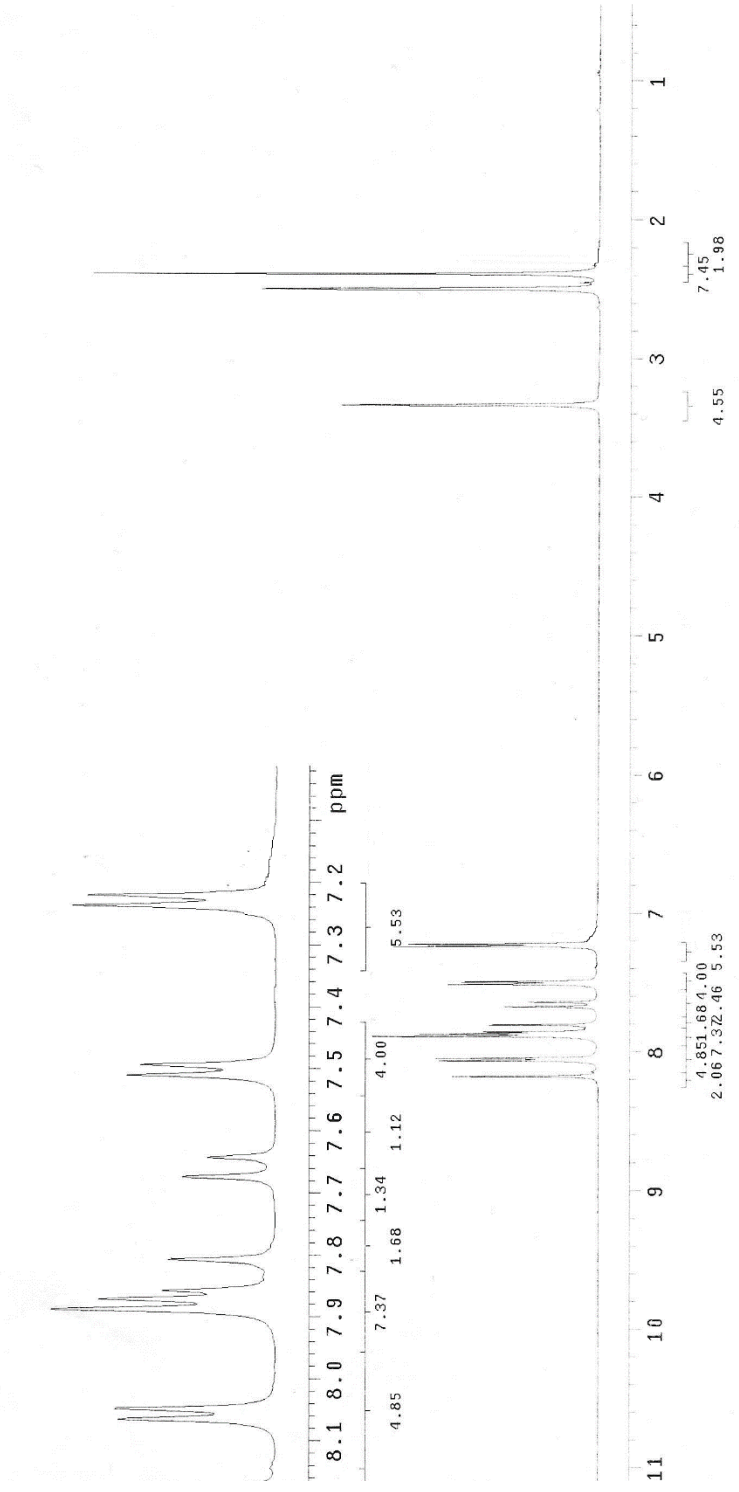


**Spectrum 8**. $^{1}$H NMR  of compd **7** (500 MHz, DMSO-$d_6$).

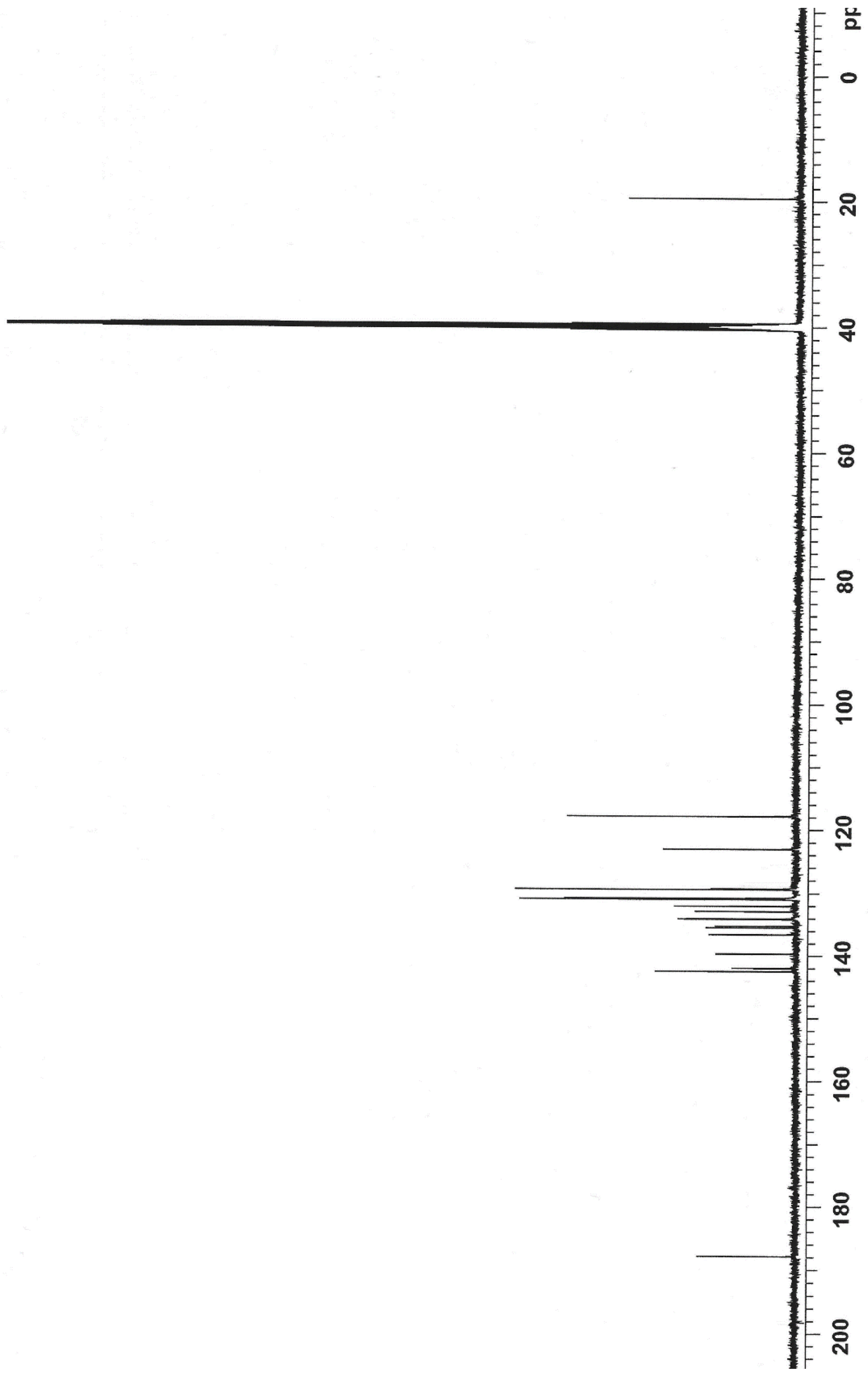


**Spectrum 9**. $^{13}$C NMR of compd **7** (125 MHz, DMSO-$d_6$).

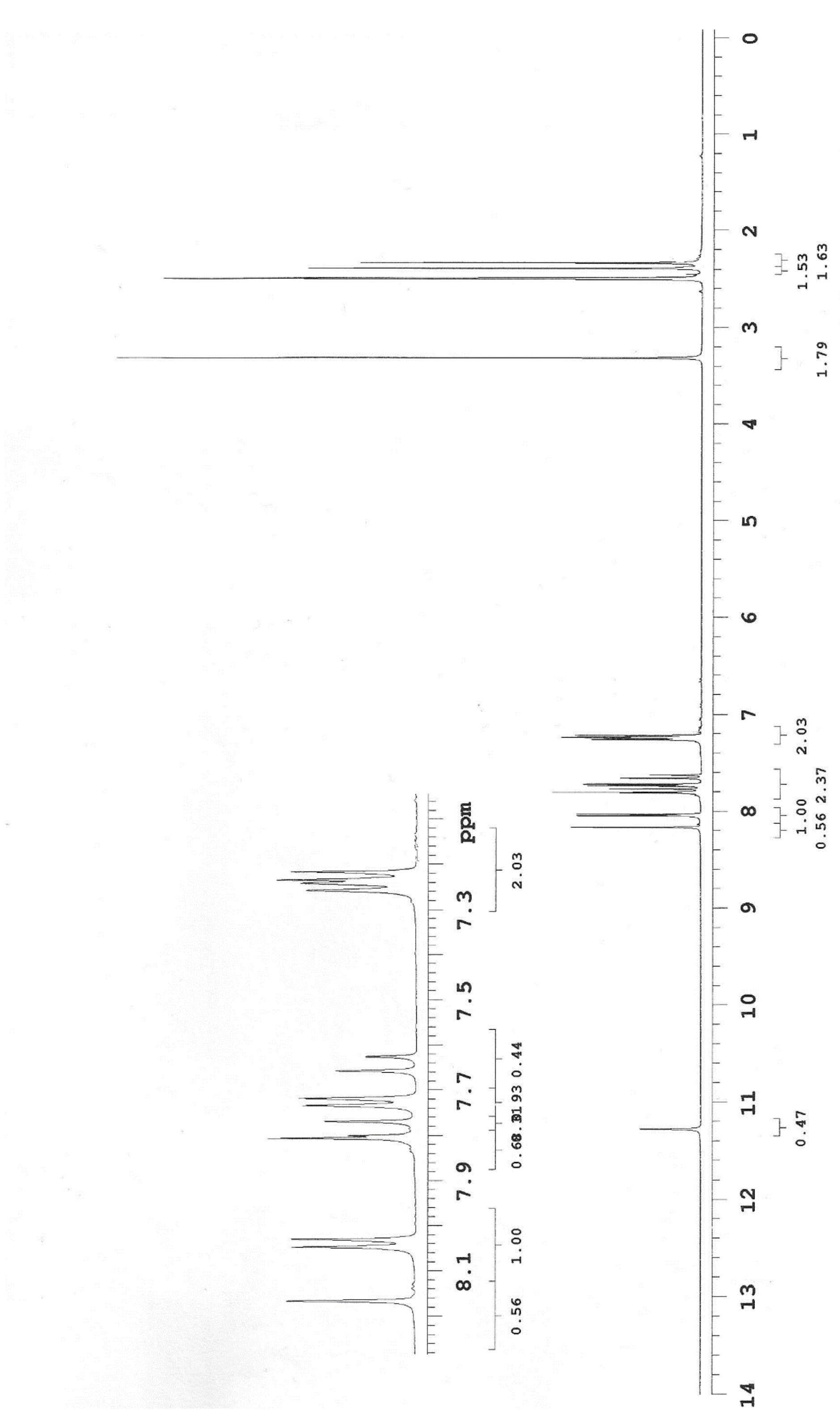


**Spectrum 10**. $^{1}$H NMR of compd **8** (500 MHz, DMSO-*d*$_6$).

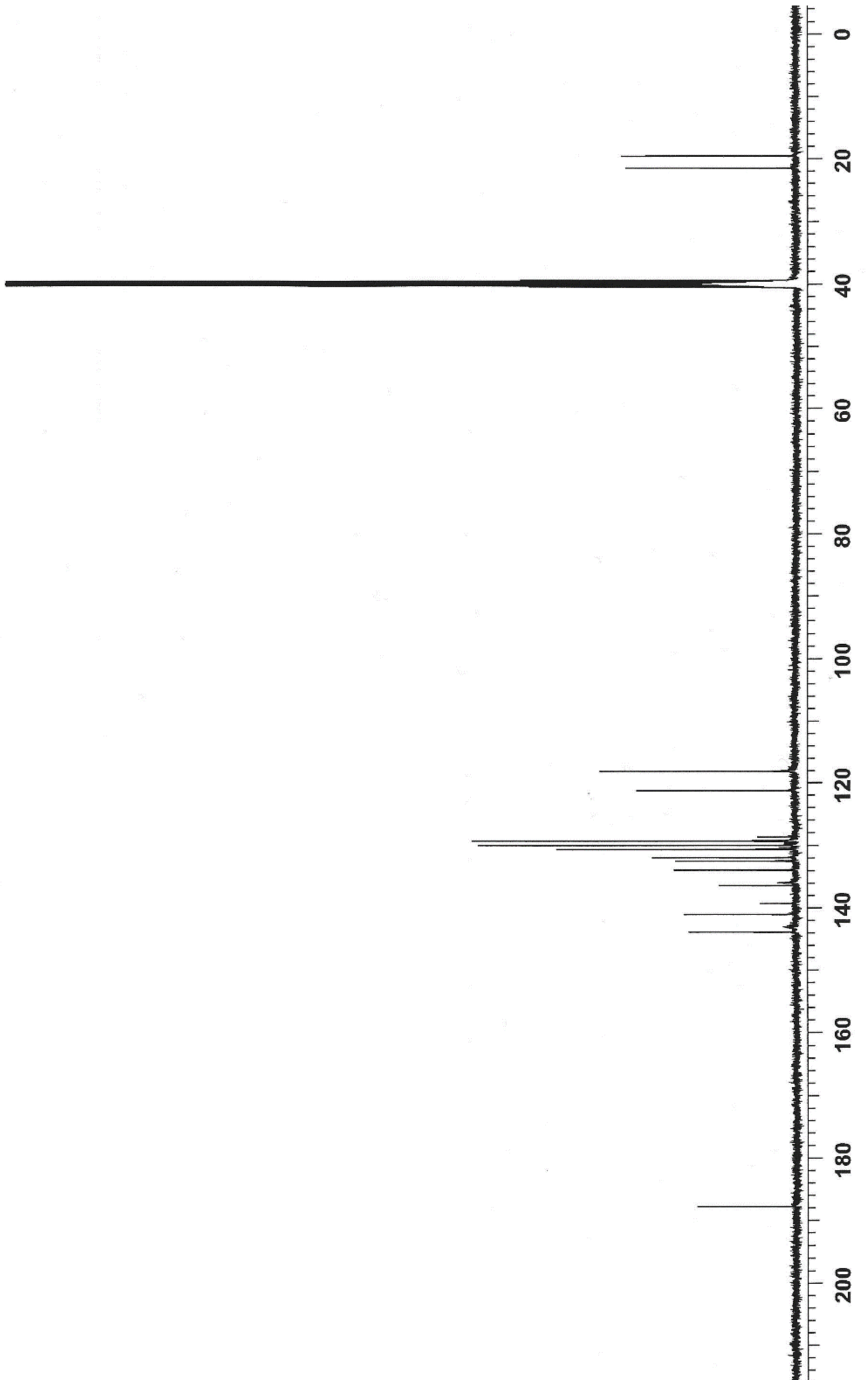


**Spectrum 11**. $^{13}C$ NMR of compd **8** (125 MHz, DMSO-$d_6$).

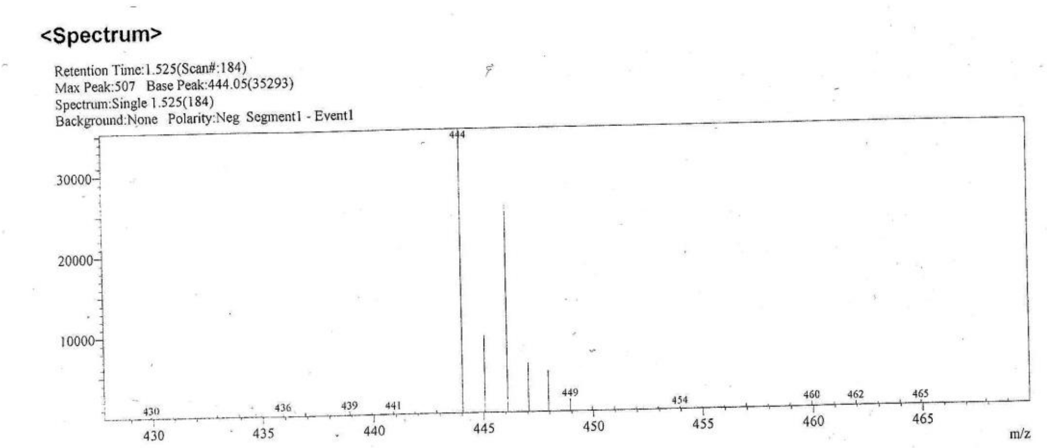


**Spectrum 12.** MS spectrum of compound **4**, MS (ESI) *m*/*z* = 444.

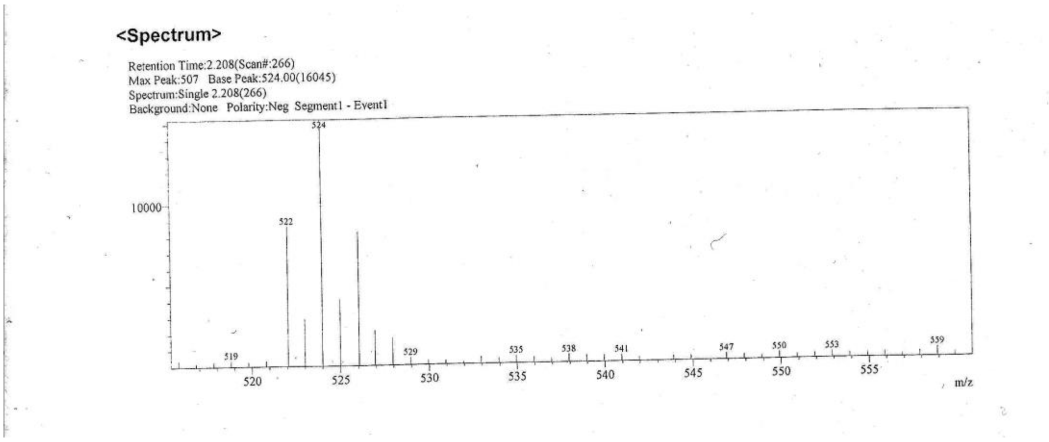


**Spectrum 13.** MS spectrum of compound **5**, MS (ESI) *m*/*z* = 522.

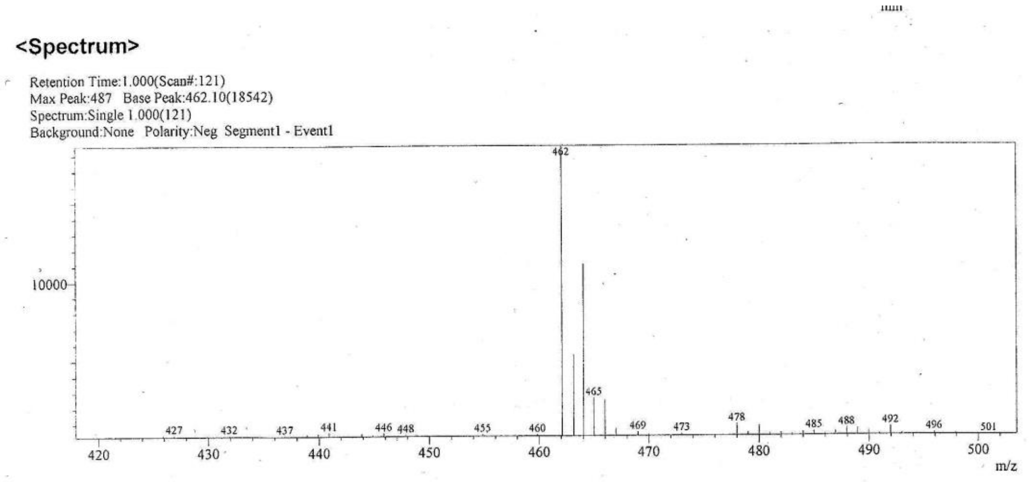


**Spectrum 14.** MS spectrum of compound **6**, MS (ESI) *m*/*z* = 462.

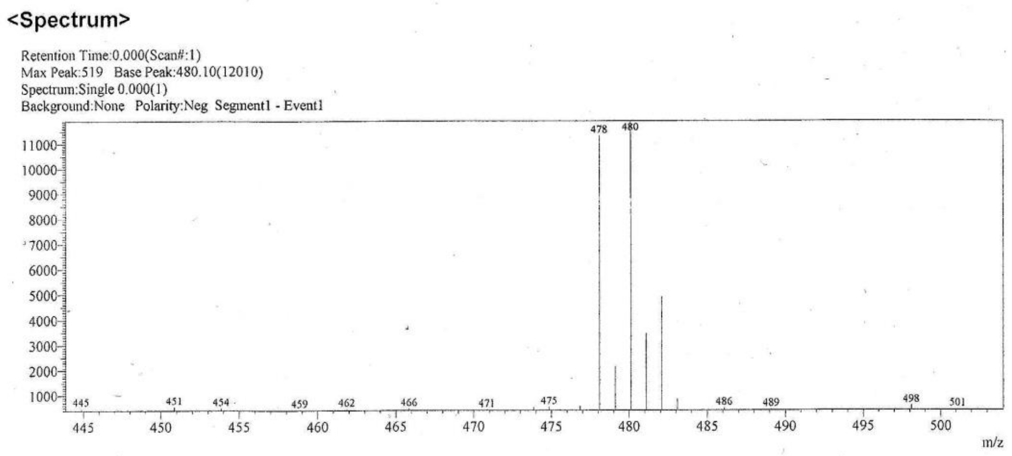


**Spectrum 15.** MS spectrum of compound **7**, MS (ESI) *m*/z = 478.

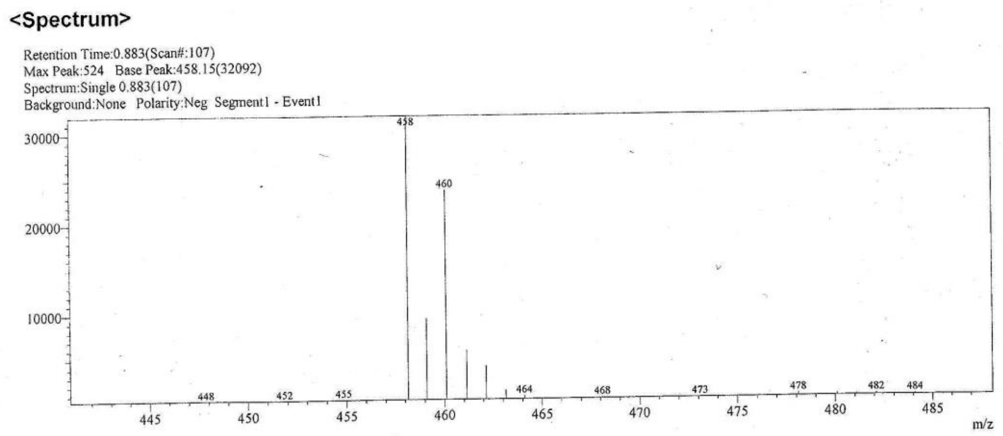


**Spectrum 16.** MS spectrum of compound **8**, MS (ESI) *m*/*z* = 458.

**Appendix B:**

**Table S1.** Minimum inhibitory concentration (MIC) [μg/mL] of derivatives on reference bacterial strains.

| **Compd** | **Minimum Inhibitory Concentration [μg/mL]** | | | | | |
|---|---|---|---|---|---|---|
| | **4** | **5** | **6** | **7** | **8** | **ampicillin** |
| *Staphylococcus epidermidis* ATCC14990 | >500 | >500 | >500 | >500 | >500 | 0.3125 |
| *Staphylococcus aureus* ATCC6538 | >500 | >500 | >500 | >500 | >500 | 0.08 |
| *Enterococcus faecalis* ATCC51299 | >500 | >500 | >500 | >500 | >500 | 0.16 |
| *Klebsiella pneumoniae* ATCC13883 | >500 | >500 | >500 | >500 | >500 | 62.5 |
| *Escherichia coli* ATCC8739 | >500 | >500 | >500 | >500 | >500 | 3.9 |
| *Salmonella enterica* ATCC13076 | >500 | >500 | >500 | >500 | >500 | 0.5 |
| *Helicobacter pylori* ATCC43504 | >500 | >500 | >500 | >500 | >500 | 3.2 |
| *Campylobacter jejuni* ZMF | >500 | >500 | >500 | >500 | >500 | 32 |
| *Campylobacter coli* ZMF | >500 | >500 | >500 | >500 | >500 | 16 |
| *Bacillus cereus* PCM 1948,2019 (ATCC11778) | >500 | >500 | >500 | >500 | >500 | 125 |
| *Listeria monocytogenes* PCM2191 | >500 | >500 | >500 | >500 | >500 | 16 |

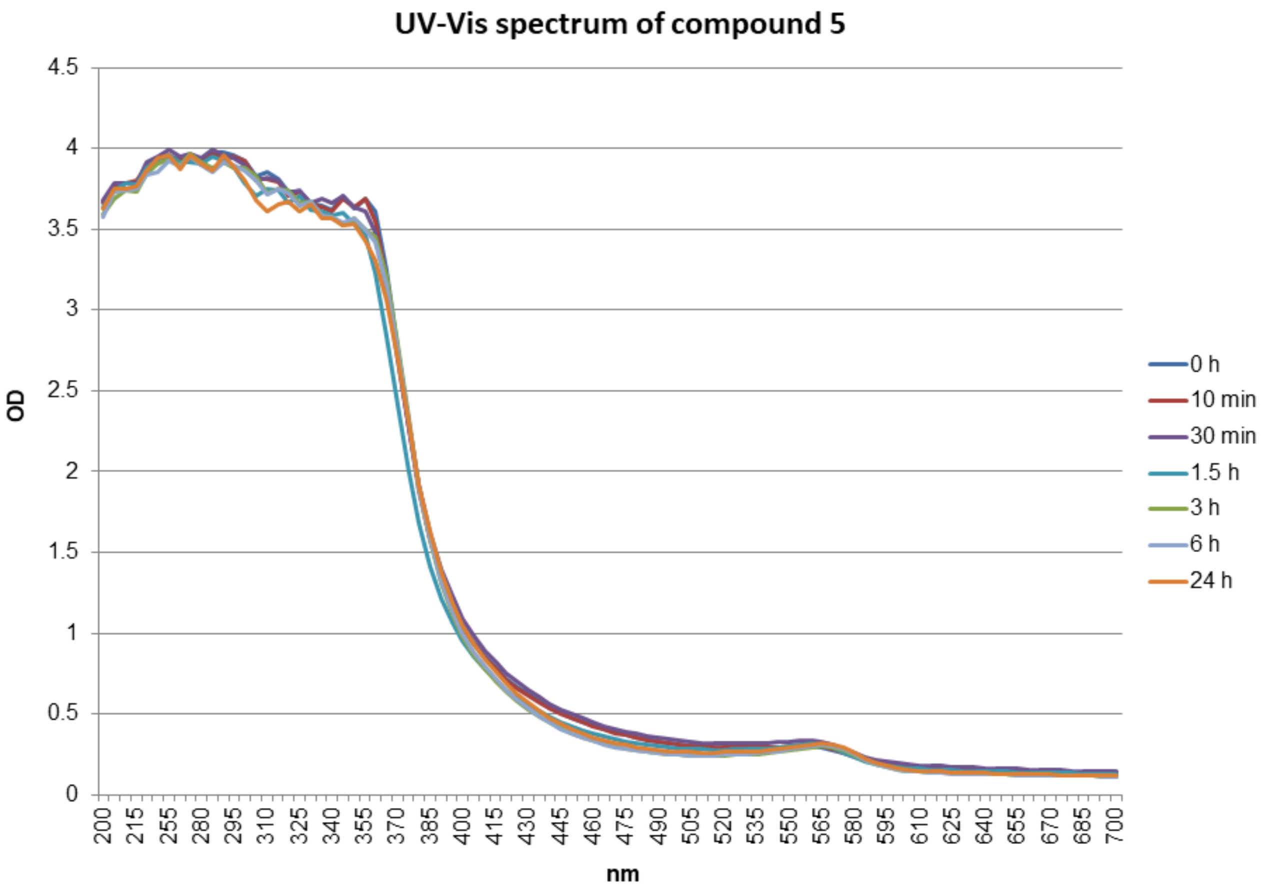


**Figure S1**. An example UV-Vis spectrum of compound 5.
